# Quetiapine-Induced Syndrome of Inappropriate Secretion of Antidiuretic Hormone

**DOI:** 10.1155/2016/4803132

**Published:** 2016-02-29

**Authors:** Theocharis Koufakis

**Affiliations:** Department of Internal Medicine, General Hospital of Larissa, 1 Tsakalof Street, 41221 Larissa, Greece

## Abstract

The syndrome of inappropriate secretion of antidiuretic hormone (SIADH) can be induced by various conditions, including malignant neoplasms, infections, central nervous system disorders, and numerous drugs. We here report a case of a 65-year-old female patient, treated with quetiapine for schizophrenia, who presented with generalized tonic-clonic seizures and was finally diagnosed with quetiapine-induced SIADH. Quetiapine-associated hyponatremia is extremely uncommon and only a few, relevant reports can be found in the literature. This case underlines the fact that patients on antipsychotic medication and more specifically on quetiapine should be closely monitored and routinely tested for electrolyte disorders.

## 1. Introduction

The syndrome of inappropriate secretion of antidiuretic hormone (SIADH) is defined by hypotonic hyponatremia, inappropriately elevated urine osmolality relative to plasma osmolality, an elevated urine sodium level, expanded extracellular volume, and normal renal, adrenal, and thyroid function [1]. SIADH can be induced by various conditions, including malignant neoplasms, infections (especially pulmonary ones), central nervous system disorders, and numerous drugs [2].

Quetiapine is an antipsychotic agent, widely used for the treatment of schizophrenia, bipolar disorder, and major depressive disorder. Frequently reported side effects of the drug include dizziness, dry mouth, nausea, constipation, lethargy, and increased appetite. Prolonged QT interval [3] and hematological effects [4] have been also described as unusual adverse reactions of quetiapine use. Still, quetiapine-associated hyponatremia is generally uncommon and only a few, relevant reports can be found in the literature.

## 2. Case Report

A 65-year-old female patient presented to the Emergency Department with generalized tonic-clonic seizures. She had been diagnosed with schizophrenia at the age of 40 and she was on quetiapine (300 mg daily, orally, divided into 3 doses), since 3 months. She denied consumption of any other drug, dry mouth symptom, and present or past history of excessive water drinking. The latter was also documented by interviewing patient's close relatives. Other than schizophrenia, her medical history was unremarkable for chronic diseases.

The patient was normotensive (blood pressure 130/85 mmHg). Physical examination did not reveal any abnormal findings. Peripheral oedema was absent. Her main laboratory findings on admission were as follows: serum sodium concentration 108 mmol/L (135–145 mmol/L), serum osmolarity 243 mOsm/L (275–295 mOsm/L), urine sodium concentration 68 mmol/L (<20 mmol/L), and urine osmolality 264 mOsm/kg. Renal, liver, and thyroid function tests as well as cortisol levels proved to be within the normal limits.

In view of these findings, the diagnosis of SIADH was established, according to the criteria described by Bartter and Schwartz [1] (Table 1). During her hospitalization, a complete diagnostic workup was performed, including thorough laboratory testing, brain, chest, and abdomen CT scans and gastrointestinal endoscopy. The above diagnostic procedures excluded other factors as the potential causes of the syndrome, such as malignancies, infections, and stroke.

Seizures were attributed to severe hyponatremia and were treated with diazepam. Initial management of the patient at the Emergency Department included intravenous infusion of 150 mL of 3% hypertonic saline (NaCl) solution over 20 minutes. After repeating the same procedure over the next 20 minutes, serum sodium concentration was measured again and found to be 113 mmol/L. Subsequently, fluids limitation (500 mL 0.9% saline daily) and quetiapine withdrawal resulted in the restoration of serum sodium concentration and plasma osmolarity to the normal levels, within the next 72 hours (135 mmol/L and 285 mOsm/L, resp.). The patient was discharged on olanzapine and, in her follow-up visits, she remained in good physical condition and her blood tests were all within the normal range.

## 3. Discussion

A wide variety of drugs has been previously accused of inducing SIADH, especially carbamazepine, selective serotonin reuptake inhibitors (SSRIs), and phenothiazines [5]. Both the newer atypical antipsychotics and the older drugs have been associated with the development of the syndrome [6]. The exact pathophysiological background of drug-induced SIADH is still unclear. However, stimulation of ADH release and increase of ADH renal action are believed to be the most probable mechanisms [2].

In most cases of SIADH associated with drugs, patients have mild, asymptomatic hyponatremia [2], which is usually detected once blood tests are ordered for an apparently irrelevant reason. However, several deaths related to hyponatremia induced by ecstasy (methylenedioxymethamphetamine or MDMA) have been reported. Moreover, cyclophosphamide- and carbamazepine-induced SIADH have been linked with high mortality [7]. In the reported case, the patient's life is endangered due to seizures triggered by severe hyponatremia.

Management of drug-induced SIADH primarily demands the discontinuation of the suspected agent. In cases of severe hyponatremia, additional actions such as fluid restriction and furosemide administration should be taken [2]. Agents causing nephrogenic diabetes insipidus, such as demeclocycline and lithium carbonate, have been also widely used for the treatment of the syndrome.

In the described case, our initial approach was treating patient with prompt infusion of hypertonic 3% saline solution, due to extremely low sodium serum levels and severe neurological symptomatology. After a 5 mmol/L increase of serum sodium concentration and stabilization of patient's neurological status, we continued with a diagnosis-specific, more conservative approach regarding the correction of sodium levels. Our decision was based, firstly, on our suspicion that SIADH and quetiapine were associated and, secondly, on our belief that patient's hyponatremia had been established more than 48 hours ago (“chronic” hyponatremia), given that the patient was on quetiapine for the past three months. It is generally accepted that symptomatic patients with severe hyponatremia should be treated aggressively to reduce cerebral edema and avoid herniation. However, rapid correction of serum sodium can result in central pontine myelinolysis and severe, irreversible neurological complications, such as spastic quadriparesis. Patients with chronic hyponatremia are at greater risk from rapid sodium correction, when compared with those with recently (<48 hours) established hyponatremia [8].

Another common cause of hyponatremia in schizophrenic patients is psychogenic polydipsia. Its distinction from SIADH should be based on history of excessive water consumption and opposite results of laboratory tests, such as urine osmolality and urine sodium levels [9].

Quetiapine-induced SIADH is extremely uncommon, as concluded from the few relevant reports found in the medical literature. Still, the exact prevalence of the above association is practically unknown. Moreover, the possibility of underdiagnosis and underreporting of this condition cannot be excluded. To our best knowledge, this is the third report in the literature, following the cases described by Atalay et al. [10] and van den Heuvel et al. [11]. Worth pointing out is the fact that, in both cases, serum sodium levels of the patients were significantly higher than the value measured in our case (128 and 120 mmol/L, resp.). In conclusion, physicians should be aware of this rare, still severe adverse reaction of quetiapine. Patients on antipsychotic medication and more specifically on quetiapine should be closely monitored and routinely tested for electrolyte disorders.

## Figures and Tables

**Table 1:** The criteria required for the diagnosis of SIADH and the main clinical and laboratory findings of the presented case, which led to the diagnosis of the syndrome.

Criteria needed for SIADH diagnosis	Patient's laboratory and clinical findings
(1) Decreased plasma osmolality (<275 mOsm/kg)	Plasma osmolarity 243 mOsm/L (275–295 mOsm/L)
(2) Inappropriately concentrated urine (>100 mOsm/kg)	Urine osmolality 264 mOsm/kg
(3) Being euvolemic	The patient was normotensive (blood pressure 130/85 mmHg). Physical examination did not reveal any abnormal findings. Peripheral oedema was absent
(4) Elevated urine Na (>20 mEq/L)	Urine Na concentration 68 mEq/L
(5) Euthyroid, eucortisolemic, and no diuretic use.	Renal, liver, and thyroid function tests and cortisol levels were within the normal limits. Comedication was not present. Patient's medical history was unremarkable for chronic diseases
